# Oral Health in Residential Settings: A Public Health Priority

**DOI:** 10.12688/hrbopenres.14265.1

**Published:** 2025-11-17

**Authors:** Roger O'Sullivan, Emma Hunter, Ruth D. Neill, Louise Bradley

**Affiliations:** 1 Institute of Public Health, Dublin, D08 NH90, Ireland; 2 The Bamford Centre, Ulster University, Coleraine, BT52 1SA, UK

**Keywords:** Residential settings, dental research, oral health, older people, interventions

## Abstract

Poor oral health is associated with increased mortality and chronic conditions. Ageing remains an unequal experience, particularly in the area of oral health. Evidence-based interventions for oral health in residential settings are limited, posing a significant public health challenge that warrants greater attention. This commentary highlights the importance of oral health and oral health research within the residential care setting, especially the need for increased research on oral health interventions.

## Introduction

The recent article by Doshi *et al*. (2025) sheds light on the important issue of oral health for adults in residential settings. As a team of public health professionals, we recently aimed to conduct an umbrella review of oral health interventions for older adults in residential settings. However, there was insufficient high-quality evidence in this area to conduct a thorough review; therefore, we reflected on why this may be the case, synthesised what is known and identified key challenges that need to be addressed for research to move forward in addressing this significant public health challenge.

One of the greatest successes of public health is that many more people are living longer than ever before; however, disparities in ageing persist, and this is particularly evident in the area of oral health. Poor oral health has been linked with a greater risk of all-cause mortality and chronic conditions (stroke, diabetes, respiratory and cardiovascular diseases) ( Bakker *et al.*, 2024; Li *et al.*, 2024). A recent systematic review of oral health outcomes among care home residents in Europe identified a high prevalence of oral health needs, including suboptimal oral hygiene, dental caries, and poor periodontal health ( Janssens *et al.*, 2023). Globally it is estimated that direct costs of $387 billion per year is attributed to oral health diseases ( Jevdjevic & Listl, 2025), and in the UK, this cost is estimated to be around £3.6 billion per year ( Office for Health Improvement and Disparities, 2022).

While there have been positive developments in population oral health, the prevalence of oral health diseases remains high among older adults, with nearly five in ten suffering from an oral health condition aged 65 and above ( World Health Organization, 2024). In particular, oral healthcare in dependent older adults in residential settings requires a greater focus and recognition of the health, access, and operational challenges ( Janssens *et al.*, 2025). Most dental diseases can be prevented, and while a wealth of evidence-based oral health interventions exists, high-quality published research to support evidence-informed interventions specifically for older adults in residential settings remains limited.

## Oral health in the residential setting

Those in residential settings are often older adults with physical or cognitive impairments who will face significant challenges, including with their oral health due to functional decline, regular barriers in access to routine dental services and supports and concurrent use of multiple medications ( Desai & Nair, 2023; Patel *et al.*, 2021). In addition to pain, infection and difficulty eating, poor oral health has also been linked to adverse general health outcomes including challenges with diabetic control and aspiration pneumonia, one of the leading causes of death in residential settings, which is particularly evident among older adults ( Office for Health Improvement and Disparities, 2022). Evidence shows that in community settings, compared to fully dentate adults, edentulous adults were more likely to have depression, experience loneliness and have reduced quality of life ( Sheehan *et al.*, 2017).

## Research challenges to oral health intervention in residential settings

Oral health interventions in older people in residential facilities are an under-researched area. This may be due to the various challenges encountered when implementing oral health interventions, including methodological considerations, access for researchers, practicalities, ethics, logistics, behavioural, and contextual factors ( Nocivelli *et al.*, 2023). Impediments to conducting interventional studies in residential settings are outlined in a recently published randomised feasibility study ( Tsakos *et al.*, 2025) and include consent issues for individuals experiencing cognitive impairment, high levels of participant attrition, and the capacity of residential settings to participate. It is also suggested that physical limitations, low health literacy and medical complexity can also play a role in the lack of studies in this cohort. Furthermore, the COVID-19 pandemic had a significant impact on oral research in residential settings, creating accessibility issues for older adults and altering the way dental professionals worked with this cohort. However, these issues are not insurmountable, and robust research has the potential to improve the quality of life for the ageing population living in residential care.

## Implications and future research

The World Health Organization (2024) launched a global oral health action plan in 2023, calling for research to address evidence gaps in public health interventions and oral health promotion in strategic settings, including the challenges of oral health in older adults residing in care facilities. Existing evidence supports the need to increase oral health training and education for staff, particularly for mouth care, proper toothbrushing and denture care, to improve oral health outcomes in those living in residential care ( Care Quality Commission, 2025a; Doshi *et al.*, 2025). In particular, there is a need for agreement on practical and ethical solutions to include residents who are unable to provide informed consent ( Tsakos *et al.*, 2025).

It is recognised that there can be significant organisational barriers for residential care settings taking part in oral health research. Research has highlighted that effective care home management, staff development and training, and a commitment to research studies are key success factors and those with a greater focus on oral health care plans ( Care Quality Commission, 2025b). Tsakos *et al.* (2025) demonstrated that co-designed interventions can be utilised to deliver consistent oral healthcare to older adults in residential settings; however, further research, particularly trials, is needed. Future work should prioritise the development of recruitment and ethics protocols suitable for all older adults, age-appropriate outcome measures, and longitudinal co-designed studies with key stakeholders to support real-world relevance.

## Conclusion

In summary, oral health interventions in residential settings represent an essential but often overlooked population. The need for high-quality evidence on oral health interventions for older people in residential settings, including multidisciplinary research teams, to inform key practices, policies, and strategies in older people's oral health, is a public health priority.

## Institutional review board statement

Not applicable.

## Informed consent statement

Not applicable.

## Data Availability

This study did not create or analyse new data, and data sharing does not apply to this article.
