# Supplementary material for: Oral Health in Residential Settings: A Public Health Priority
Source: HRB Open Res. 2026 Feb 11;8:120. Originally published 2025 Nov 17. [Version 2] doi: 10.12688/hrbopenres.14265.2 (PMC12949375; doi:10.12688/hrbopenres.14265.2)
Supplement: Peer review history

# Reviewer report: Reviewer response for version 2

**DOI:** 10.21956/hrbopenres.15800.r53440

**Published:** 2026-02-27

**Authors:** Khaing Myat Thu

**Affiliations:** 1 The University of Hong Kong, Hong Kong, China

The author has addressed the comments. No further questions.

Are arguments sufficiently supported by evidence from the published literature or by new data and results?

Yes

Is the conclusion balanced and justified on the basis of the presented arguments?

Yes

Is the rationale for commenting on the previous publication clearly described?

Partly

Are any opinions stated well-argued, clear and cogent?

Yes

Reviewer Expertise:

Geriatric Dentistry, Special Care Dentistry, Artificial Intelligence, Removable Prosthodontics

I confirm that I have read this submission and believe that I have an appropriate level of expertise to confirm that it is of an acceptable scientific standard.

# Reviewer report: Reviewer response for version 1

**DOI:** 10.21956/hrbopenres.15689.r51850

**Published:** 2026-01-02

**Authors:** Archana Pradhan

**Affiliations:** 1 University of Sydney, Sydney, Australia

**Summary**

 The commentary is well-written addressing the critical issue of oral health among older adults in residential care settings. It synthesizes key evidence gaps and challenges, and it directly aligns the discussion with recent global initiatives for older adults in residential care settings. The article would be of interest to both researchers and practitioners interested in geriatric oral health.

 I have a few comments and suggestions as below:

**Abstract**

 Evidence-based interventions for oral health **exist, however are not implemented** in residential settings and warrants greater attention. Rather than emphasis on the need for increased research on oral health interventions, which is acknowledged as being challenging, the focus should be on the implementation of evidenced-based preventive and minimal intervention dentistry onsite and the cost-effectiveness, acceptability and sustainability of such programs.

**Introduction**

 The authors reflected on why there is insufficient high-quality evidence, synthesised and identified key challenges to address this significant public health issue. They have referred to recent systematic review by Janssens *et al.*, 2023 who identified a high prevalence of oral health needs, including suboptimal oral hygiene, dental caries, and poor periodontal health among care home residents in Europe. However, tooth wear (Chan *et al. *2024) and denture related problems (Taira *et al. *2023 *, *Mameno *et al.* 2023) have been overlooked.

**Oral health in the residential setting**

 Under this topic, the **adverse effects of oral health** have been stated, rather than oral health problems.

**Research challenges to oral health intervention in residential settings**

 Most challenges implementing oral health interventions, including ethics, consent, methodological considerations, behavioural issues for people with cognitive impairment, high levels of participant attrition, medical complexity and physical limitations, low health literacy with additional impacts from the COVID-19 pandemic on accessibility and dental services were discussed. The **robust research **needed to improve the quality of life for the ageing population living in residential care was suggested but not specified.

**Implications and future research**

 It is important to increase oral health training and education for staff for mouth care, toothbrushing and denture care, to improve oral health outcomes in those living in residential care. In addition,

 It is important to note that dental practitioners themselves lack confidence in providing dental care to older people, especially those with cognitive impairment due to inadequate training and experience and yet many do not take advantage of the online training made available/accessible to them (Pradhan and Lalloo 2021). inclusion of Geriatric dentistry in dental education is therefore urgently needed to prepare the dental workforce to meet the complex dental treatment needs of the growing ageing population (Nilsson *et al.* 2019).

Are arguments sufficiently supported by evidence from the published literature or by new data and results?

Partly

Is the conclusion balanced and justified on the basis of the presented arguments?

Partly

Is the rationale for commenting on the previous publication clearly described?

Yes

Are any opinions stated well-argued, clear and cogent?

Partly

Reviewer Expertise:

Special Needs Dentistry and Gerodontology

I confirm that I have read this submission and believe that I have an appropriate level of expertise to confirm that it is of an acceptable scientific standard.

# Response

**Published:** 2026-01-30

**Authors:** Ruth Neill

**Affiliations:** Institute of Public Health, Not Applicable, Belfast, Ulster, Ireland

**Author response to reviewer comments.**

**Summary**

 The commentary is well-written addressing the critical issue of oral health among older adults in residential care settings. It synthesizes key evidence gaps and challenges, and it directly aligns the discussion with recent global initiatives for older adults in residential care settings. The article would be of interest to both researchers and practitioners interested in geriatric oral health.

 I have a few comments and suggestions as below:

 Author comment: The authors would like to thank the reviewer for their time in reviewing the manuscript.

**Abstract**

 Evidence-based interventions for oral health **exist, however are not implemented** in residential settings and warrants greater attention. Rather than emphasis on the need for increased research on oral health interventions, which is acknowledged as being challenging, the focus should be on the implementation of evidenced-based preventive and minimal intervention dentistry onsite and the cost-effectiveness, acceptability and sustainability of such programs.

 Author comment: The authors have amended the text in the section: *Abstract to address the reviewers' comments *to highlight the gap in the research and the need for more evidence based practice.

**Introduction**

 The authors reflected on why there is insufficient high-quality evidence, synthesised and identified key challenges to address this significant public health issue. They have referred to recent systematic review by Janssens *et al.*, 2023 who identified a high prevalence of oral health needs, including suboptimal oral hygiene, dental caries, and poor periodontal health among care home residents in Europe. However, tooth wear (Chan *et al. *2024) and denture related problems (Taira *et al. *2023 *, *Mameno *et al.* 2023) have been overlooked.

 Author comment: The authors have amended the text in the section: *Introduction *to include the new references and addressed the reviewer’s comments.

**Oral health in the residential setting**

 Under this topic, the**** **adverse effects of oral health** have been stated, rather than oral health problems.

 Author comment: The authors have amended the text in the section on oral health in residential settings to address the reviewers' comments and to discuss oral health problems and their adverse effects.​​​​​​​

**Research challenges to oral health intervention in residential settings**

 Most challenges implementing oral health interventions, including ethics, consent, methodological considerations, behavioural issues for people with cognitive impairment, high levels of participant attrition, medical complexity and physical limitations, low health literacy with additional impacts from the COVID-19 pandemic on accessibility and dental services were discussed. The **robust research **needed to improve the quality of life for the ageing population living in residential care was suggested but not specified.

 Author comment: ​​​​​​​The authors have amended the text in the section: *research challenges to oral health intervention in residential settings to address the reviewers' comments *and to highlight some of the methodological considerations that are important challenges in oral health care research.​​​​​​​

**Implications and future research**

 It is important to increase oral health training and education for staff for mouth care, toothbrushing and denture care, to improve oral health outcomes in those living in residential care. In addition,

 It is important to note that dental practitioners themselves lack confidence in providing dental care to older people, especially those with cognitive impairment due to inadequate training and experience and yet many do not take advantage of the online training made available/accessible to them (Pradhan and Lalloo 2021). inclusion of Geriatric dentistry in dental education is therefore urgently needed to prepare the dental workforce to meet the complex dental treatment needs of the growing ageing population (Nilsson *et al.* 2019).

 Author comment: The authors have amended the text in the section: *implications and future research *to address the reviewers' comments and add the suggested references.

# Reviewer report: Reviewer response for version 1

**DOI:** 10.21956/hrbopenres.15689.r51851

**Published:** 2025-12-26

**Authors:** Khaing Myat Thu

**Affiliations:** 1 The University of Hong Kong, Hong Kong, China

This commentary effectively highlights the research gap in oral health within residential aged-care facilities, an area that remains challenging for oral health researchers.

1. To strengthen the piece, please consider explicitly noting that most studies in these settings are cross-sectional and briefly explaining why trials or longitudinal studies are uncommon.
2. Participation and willingness of facilities, staff, and residents, as research activities can add burden beyond their daily routines. Please discuss strategies to minimize burden and enhance engagement.
3. As the qualitative research is crucial to understand how administrators and care teams perceive the importance and feasibility of routine oral care, please consider to discuss it.

These additions would sharpen the articulation of current research needs and provide clearer guidance for future work.

 Given the commentary’s aim to frame this as a public health priority, please also consider including:

1. National policy and government support: how evidence can inform policy design and how the policy shape the oral healthcare in residential setting
2. Caregiver-to-resident ratios from an oral care perspective: the imbalanced ratios increase caregiver burden and may crowd out oral care tasks.
3. The role of caregivers in oral care: as the caregivers are key for daily oral care of frail and disabled residents, highlight the need for their motivation, competency-based training and support.

Are arguments sufficiently supported by evidence from the published literature or by new data and results?

Yes

Is the conclusion balanced and justified on the basis of the presented arguments?

Yes

Is the rationale for commenting on the previous publication clearly described?

Partly

Are any opinions stated well-argued, clear and cogent?

Yes

Reviewer Expertise:

Geriatric Dentistry, Special Care Dentistry, Artificial Intelligence, Removable Prosthodontics

I confirm that I have read this submission and believe that I have an appropriate level of expertise to confirm that it is of an acceptable scientific standard, however I have significant reservations, as outlined above.

# Response

**Published:** 2026-01-30

**Authors:** Ruth Neill

**Affiliations:** Institute of Public Health, Not Applicable, Belfast, Ulster, Ireland

**Author responses to reviewer comments.**

 This commentary effectively highlights the research gap in oral health within residential aged-care facilities, an area that remains challenging for oral health researchers.

 Author comment: The authors would like to thank the reviewer for taking the time to review the manuscript.

 1. To strengthen the piece, please consider explicitly noting that most studies in these settings are cross-sectional and briefly explaining why trials or longitudinal studies are uncommon.

 Author comment:The authors have added new text to the section titled: *Research challenges to oral health intervention in residential settings *to highlight the different types of studies.

 2. Participation and willingness of facilities, staff, and residents, as research activities can add burden beyond their daily routines. Please discuss strategies to minimize burden and enhance engagement.

 Author comment: The authors have added new text to the section titled: *Research challenges to oral health intervention in residential settings*, to challenges to research in this setting, and how these challenges could be minimised.

 3. As the qualitative research is crucial to understand how administrators and care teams perceive the importance and feasibility of routine oral care, please consider to discuss it.

 Author comment: The authors have amended the Implications and future research section to address the reviewers' comments on the importance of future qualitative studies.

 These additions would sharpen the articulation of current research needs and provide clearer guidance for future work.

 Given the commentary’s aim to frame this as a public health priority, please also consider including

 1. National policy and government support: how evidence can inform policy design and how the policy shape the oral healthcare in residential setting.

 Author comment: The authors have amended the text within the section titled: *Implications and future research *to highlight public health policy.

 2. Caregiver-to-resident ratios from an oral care perspective: the imbalanced ratios increase caregiver burden and may crowd out oral care tasks.

 Author comment: The authors have amended the section titled "Implications and future research" to highlight staff-to-resident ratios.

 3. The role of caregivers in oral care: as the caregivers are key for daily oral care of frail and disabled residents, highlight the need for their motivation, competency-based training and support.

 Author comment: The authors have amended the text within the section titled: *Implications and future research *to highlight the need to improve and utilise training and education pathways for staff.
